# Ocean warming and acidification modulate energy budget and gill ion regulatory mechanisms in Atlantic cod (*Gadus morhua*)

**DOI:** 10.1007/s00360-015-0923-7

**Published:** 2015-07-29

**Authors:** C. M. Kreiss, K. Michael, M. Lucassen, F. Jutfelt, R. Motyka, S. Dupont, H. -O. Pörtner

**Affiliations:** Alfred Wegener Institute, Helmholtz Center for Marine and Polar Research, Integrative Ecophysiology, Am Handelshafen 12, 27570 Bremerhaven, Germany; Department of Biological and Environmental Sciences, University of Gothenburg, PO Box 463, 405 30 Gothenburg, Sweden; The Sven Lovén Centre for Marine Sciences, Kristineberg 566, 451 78 Fiskebäckskil, Sweden

**Keywords:** Na^+^/K^+^-ATPase, H^+^-ATPase, HCO_3_^−^ transporter, Na^+^/H^+^-exchanger, Standard metabolic rate, Osmolality

## Abstract

Ocean warming and acidification are threatening marine ecosystems. In marine animals, acidification is thought to enhance ion regulatory costs and thereby baseline energy demand, while elevated temperature also increases baseline metabolic rate. Here we investigated standard metabolic rates (SMR) and plasma parameters of Atlantic cod (*Gadus morhua*) after 3–4 weeks of exposure to ambient and future *P*CO_2_ levels (550, 1200 and 2200 µatm) and at two temperatures (10, 18 °C). *In vivo* branchial ion regulatory costs were studied in isolated, perfused gill preparations. Animals reared at 18 °C responded to increasing CO_2_ by elevating SMR, in contrast to specimens at 10 °C. Isolated gills at 10 °C and elevated *P*CO_2_ (≥1200 µatm) displayed increased soft tissue mass, in parallel to increased gill oxygen demand, indicating an increased fraction of gill in whole animal energy budget. Altered gill size was not found at 18 °C, where a shift in the use of ion regulation mechanisms occurred towards enhanced Na^+^/H^+^-exchange and HCO_3_^−^ transport at high *P*CO_2_ (2200 µatm), paralleled by higher Na^+^/K^+^-ATPase activities. This shift did not affect total gill energy consumption leaving whole animal energy budget unaffected. Higher Na^+^/K^+^-ATPase activities in the warmth might have compensated for enhanced branchial permeability and led to reduced plasma Na^+^ and/or Cl^−^ concentrations and slightly lowered osmolalities seen at 18 °C and 550 or 2200 µatm *P*CO_2_ in vivo. Overall, the gill as a key ion regulation organ seems to be highly effective in supporting the resilience of cod to effects of ocean warming and acidification.

## Introduction

Anthropogenic climate change has profound impacts on marine ecosystems as the oceans become warmer and are acidified by the uptake of atmospheric carbon dioxide (Pörtner et al. 2014). Depending on emission scenario atmospheric *P*CO_2_ levels are projected to reach between 420 and 940 µatm by the year 2100, consistent with an average decrease in surface ocean pH by 0.13–0.42 units. Most of the ocean will continue to warm although rates vary regionally and differ highly between emission scenarios (Collins et al. 2013). Initial studies projected that highly mobile organisms such as marine fishes are excellent osmotic and acid–base regulators and therefore better able to cope with acidification than e.g. more inactive invertebrates with lower ion regulation capacity (Melzner et al. 2009; Wittmann and Pörtner 2013). More recent behavioural studies, mainly of coral reef fish larvae have suggested that some fishes may still be very sensitive when ambient CO_2_ increases (e.g. Munday et al. 2012; Nilsson et al. 2012; Forsgren et al. 2013). Behavioural effects on sensitive fish species set in at rather low concentrations (<850 µatm CO_2_); however, the long-term persistence of these phenomena remains to be explored (Wittmann and Pörtner 2013). In fact, Atlantic cod appears resilient to behavioural disturbances at CO_2_ levels around 1000 µatm (Jutfelt and Hedgärde 2013) and up to 4200 µatm (Maneja et al. 2013). In juvenile cod, aerobic swimming performance was unaffected by exposure to high *P*CO_2_ (3000 and 6000 µatm) at close to optimum temperatures (Melzner et al. 2009). However, larval stages may be more sensitive. Severe tissue damage was reported for a fraction of the larval offspring of Norwegian coastal cod (Frommel et al. 2011). In contrast, the tissues of early larval stages of Baltic cod remained unaffected up to 3200 µatm *P*CO_2_ (Frommel et al. 2013). Nonetheless, Atlantic cod avoid hypercapnic water, indicating that elevated *P*CO_2_ may have unfavourable effects (Jutfelt and Hedgärde 2013).

More is known about the sensitivity of fish including Atlantic cod to temperature changes (for review see Pörtner et al. 2008; Pörtner and Peck 2010). In cod, the thermal range and associated growth performance characterise populations in a latitudinal cline. The thermal window of performance is wide in juvenile fishes and shrinks with increasing body size, especially due to a weight dependent shift in thermal optima and upper thermal limits to cooler temperatures (Pörtner et al. 2008). The warm-induced decline in performance indicates thermal limitation and has been attributed to the onset of a mismatch between oxygen supply capacity and costs on the one hand and oxygen demand on the other hand, at the so-called pejus temperatures. In common eelpout (*Zoarces viviparus*) pejus limits were identified to parallel the onset of ecological effect, a loss in field abundance due to warming extremes (Pörtner and Knust 2007). Progressive warming beyond a putative critical limit of 16 °C caused a sharp drop in venous *P*O_2_ (P_V_O_2_), in parallel to the onset of cardiac arrhythmia in cannulated cod (Lannig et al. 2004; it should be noted that cannulation may enhance circulatory cost and cause a downward shift of these limits). As cardiac activity did not compensate for reduced P_V_O_2_ in the warmth, reduced oxygen supply to tissues and thus decreased aerobic scope of the whole organism above pejus limits was proposed as the first level of thermal limitation (Lannig et al. 2004; Pörtner et al. 2004). Thermal constraints may affect the scope of energy dependent mechanisms sustaining homeostasis, such as ion and acid–base regulation, and their share in energy budget, especially if challenged by ocean acidification. These changes may feedback on thermal tolerance.

Water breathers exposed to elevated *P*CO_2_ in seawater need to re-establish acid–base equilibria through proton equivalent ion exchange. In marine fish, ion regulation mainly takes place in the gills causing a net uptake of bicarbonate within 20–30 min, varying with the extent of acid–base disturbance (Evans et al. 2005). Membrane proteins, including Na^+^/H^+^-exchangers, V-type H^+^-ATPase, HCO_3_^−^-transporters and Na^+^/HCO_3_ co-transporters contribute to these processes and have associated energetic costs (e.g. Claiborne et al. 2002; Deigweiher et al. 2008; Heuer and Grosell, 2014). Many studies have focused on the identification and localization of the proteins involved in acid–base regulation applying immunological and molecular techniques, while knowledge of in vivo usage of specific transporter and associated metabolic costs is scarce. In branchial tissue, Na^+^/K^+^-ATPase is generally thought to be the key transporter driving most energy dependent ion transport processes including those related to acid–base regulation (Deigweiher et al. 2008). An upregulation of this important transport protein, determined via analyses of activity in isolated gills or of enzyme capacity in crude gill homogenates in cod, notothenioids, and eelpout gills was observed under CO_2_ concentrations of >6000 µatm; ranging from acute exposure to up to 1 year of acclimation (Deigweiher et al. 2008; Melzner et al. 2009; Deigweiher et al. 2010). In contrast, cod acclimated long term to moderately elevated *P*CO_2_ (2500 µatm) revealed reduced branchial energy turnover in vivo at unchanged in vitro Na^+^/K^+^-ATPase capacity (Kreiss et al. 2015).

Here we conducted a follow-up experiment with transporter specific inhibitors in order to identify the mechanisms contributing to acid–base regulation. We exposed Atlantic cod from the Kattegat/Skagerrak to different levels of *P*CO_2_ and temperature for 4 weeks. Atlantic cod is expected to experience strong anthropogenic climate change (Drinkwater 2005), as its habitat on the European Shelf is estimated to undergo one of the highest rates of change in seasonal temperature maxima and minima (Taboada and Anadón 2012). The North Sea region might also exceed the globally predicted average of acidification and reach 1000 µatm CO_2_ already around the year 2060 (Blackford and Gilbert 2007). In our experiments, we used 10 °C as the optimum temperature, which is close to average habitat temperature and represents the maximum growth temperature of young adults of this species (Pörtner et al. 2008). For the high temperature treatment, we chose 18 °C as this is well in the range of maximum summer values experienced by Atlantic cod (Neat and Righton 2007; Righton et al. 2010). *P*CO_2_ levels were selected to cover present and future natural variability at the study site: 550 µatm was chosen as a low value, 1200 µatm as a medium and 2200 µatm as a high CO_2_ partial pressure. Seawater *P*CO_2_ in the Atlantic in general is characterised by a seasonal cycle with surface waters being oversaturated during winter, summer and autumn, while being undersaturated during spring (Takahashi et al. 1993; Gypens et al. 2011). *P*CO_2_ also increases in parallel to seasonal temperature. We hypothesise that the combination of elevated temperature and *P*CO_2_ will affect the energy demand at whole animal level, as a consequence of shifts in ion regulation and associated energy (re)allocation. We also hypothesise that changes in branchial Na^+^/K^+^-ATPase usage under high *P*CO_2_ and/or temperature will reflect altered activities of other ion transporters and lead to altered plasma ionic status.

## Materials and methods

Experiments using live animals were approved according to ethical commission Dnr.: 23-2012, Sweden’s Ethical Committee on Animal Experiments.

### Experimental animals

Atlantic cod *Gadus morhua* of mixed gender (200.52 ± 95.4 g FW) were caught in fish traps in the Gullmarfjord around the Sven Lovén Centre for Marine Sciences—Kristineberg (Fiskebäckskil, Sweden) in February/March 2012 (permission: Dnr.: 3157-11). At the Marine Station, the fish were held for 2–3 weeks in 1200 l tanks with natural flowing seawater at 10 °C. For the experiments, fish were tagged individually (Visible Implant Elastomer, Northwest Marine Technology Inc. Washington, USA) while anaesthetized with MS-222 (3-aminobenzoic ethyl ester, Applichem, Darmstadt; Germany) at a concentration of 0.2 g/l. Afterwards animals were exposed for 4 weeks to 10.3 ± 0.2 °C and 18.1 ± 0.2 °C at 553 ± 78 µatm CO_2_, 1470 ± 460 µatm CO_2_ and 2228 ± 312 µatm CO_2_ (Table 1). These values encompassed the range of interannual *P*CO_2_ fluctuations reported for the Gullmarsfjord from ~500 to 1000 µatm, paralleled by pH oscillations of about 0.15 between 10 °C (more alkaline) and 18 °C (more acidified, Swedish Oceanographic Data Centre (SODC), pH data recorded in the Gullmarsfjord from 1921 to 1989, recalculated by S. Dupont). 8–10 fish were incubated per 1200 l tank, with two replicate tanks per treatment. Fish in the high temperature treatment were directly exposed to 18 °C without gradual warming. CO_2_-partial pressures of 1200 and 2200 µatm equivalent to pH 7.7 and 7.5 were maintained by a computerised feedback system (Aqua Medic GmbH, Bissendorf, Germany), which regulates pH (NBS scale) by addition of gaseous CO_2_ to the seawater tank (±0.02 pH units). Fish reared at low *P*CO_2_ were maintained in aerated natural seawater. To ensure a consistent mixture of the water, mass pumps with a power of 40 l/min (Eheim GmbH, Deizisau, Germany) were inserted in the tanks. The cod were fed three times a week until satiation with frozen shrimp and blue mussels and were maintained under a 12:12 day: night cycle. Animals were starved for 48 h prior to preparation and experiments. Water chemistry was controlled twice a week by measuring pH and temperature (portable pH metre Profi line pH 3310, WTW GmbH, Weilheim, Germany, NIST Scale corrected to total scale via Dickson standards). Total alkalinity was measured photometrically with an accuracy of 10 µmol/kg^−1^ seawater according to Sarazin et al. (1999). For total dissolved inorganic carbon (DIC), an autoanalyser (SFA QuAAtro 800 TM, SEAL, Wisconsin, USA) was used; salinity was measured with a conductivity metre (Cond 1970i, WTW GmbH, Weilheim, Germany). *P*CO_2_ values were calculated using the CO_2_sys programme [constants of Mehrbach et al. (1973); refitted by Dickson and Millero (1987) developed by Lewis and Wallace (1998) (Table 1)].

**Table 1:** Water parameters of different treatments during animal incubation with two replicates each (1 and 2)

Parameter	Low tank 1	Low tank 2	Medium tank 1	Medium tank 2	High tank 1	High tank 2
Temperature	10.6 ± 0.2	10.4 ± 0.2	10.3 ± 0.1	10.1 ± 0.1	10.3 ± 0.2	10.1 ± 0.1
*P*CO_2_ [µatm]	504.36 ± 54.52	488.51 ± 52.42	1151.00 ± 120.78	1111.84 ± 75.67	2184.54 ± 338.4	2131.62 ± 317.7
pH (NBS scale)	8.13 ± 0.03	8.13 ± 0.03	7.76 ± 0.02	7.74 ± 0.03	7.50 ± 0.06	7.50 ± 0.03
pH (total scale)	7.97 ± 0.04	7.98 ± 0.04	7.65 ± 0.02	7.63 ± 0.04	7.37 ± 0.06	7.38 ± 0.04
Salinity [PSU]	32.6 ± 0.3	32.6 ± 0.2	32.6 ± 0.5	32.5 ± 0.5	32.6 ± 0.2	32.6 ± 0.2
DIC [µmol/L]	2325.5 ± 65.6	2297.1 ± 42.9	2363.9 ± 31.4	2373.6 ± 66.2	2495.0 ± 90.0	2325.5 ± 65.6
Alkalinity	2326.4 ± 68.9	2337.0 ± 44.9	2330.2 ± 34.5	2327.8 ± 24.2	2329.6 ± 28.8	2327.4 ± 29.8
Temperature	17.9 ± 0.2	18.0 ± 0.1	18.2 ± 0.1	18.1 ± 0.1	18.2 ± 0.2	18.2 ± 0.1
*P*CO_2_ [µatm]	595.94 ± 59.75	623.08 ± 50.30	1278.01 ± 80.16	1305.32 ± 82.96	2366.74 ± 340.3	2214.85 ± 229.4
pH (NBS scale)	8.04 ± 0.05	8.02 ± 0.01	7.77 ± 0.02	7.76 ± 0.02	7.50 ± 0.06	7.53 ± 0.05
pH (total scale)	7.91 ± 0.03	7.89 ± 0.03	7.61 ± 0.02	7.60 ± 0.02	7.36 ± 0.06	7.39 ± 0.04
Salinity [PSU]	32.0 ± 1.2	32.0 ± 1.0	32.6 ± 0.3	32.6 ± 0.3	32.0 ± 1.1	32.1 ± 1.0
DIC [µmol/l]	2296.5 ± 58.5	2279.4 ± 44.9	2338.7 ± 4.1	2364.5 ± 66.2	2370.7 ± 62.4	2390.2 ± 81.2
Alkalinity	2326.0 ± 41.4	2315.0 ± 32.7	2326.0 ± 28.0	2334.0 ± 30.0	2324.7 ± 38.8	2326.6 ± 38.9

### Oxygen consumption of whole fish

Standard metabolic rate (MO_2min_) of Atlantic cod was measured by intermittent-flow respirometry of individual fish (six measured in parallel) starting after 3 weeks of experimental exposure of 12 fish per treatment (including six fish from each replicate tank). The fish were starved for 48 h in their exposure tanks before placing them into the respirometer, a custom-built sealed 3 l horizontal cylindrical acrylic respirometer with a circulation pump (5 l/min) (Eheim GmbH & Co., Deizisau Germany), equipped with an in-line robust 3 mm oxygen optode (FireSting, Pyroscience, Aachen, Germany). The optode was connected to the optical oxygen meter (FireStingO_2_) which in turn was connected to a PC running the Pyro Oxygen Logger (FireSting Pyroscience, Aachen, Germany). The fish were kept undisturbed in the respirometer for 48 h with continuous 20 min measurements followed by 10 min of flushing, controlled by a timer. The O_2_ saturation in the respirometers was always above 80 %. The mean of the lowest 10 % of recorded MO_2_ values is presented as standard metabolic rate, calculated as M(O_2_) = [µmol (O_2_) × gFW^−1^ × h^−1^]. During respirometry of fish at 18 °C and low *P*CO_2_, two fish died from asphyxiation due to water pump failure interrupting the flow. Whole animal as well as gill oxygen consumption rates of the remaining fish from the same tank were significantly reduced compared to the replicate tank. As we cannot preclude that the fish released chemical alarm signals in response to hypoxia (Lebedeva et al. 1994) or excreted catecholamines (Butler et al. 1989), which might induce decreased metabolism (Wahlquivst and Nilsson 1977), we excluded the respective data from further analyses. In later studies, all fish subjected to respiration protocols displayed parameters such as blood ion concentration and osmolality or those investigated in isolated gill experiments identical to those not exposed to respirometry.

### Animals and isolated gill preparation

Fish were anaesthetised with 0.2 g/l MS222 and identified by the tag. Blood samples were taken from the ventral vein for later analysis of osmolality and ionic composition. Subsequent to killing the animals by cutting their spine, gill arches were quickly dissected. The first two arches from each side of five fish (10 per treatment and replicate) were used for experiments and immediately placed in ice-cold saline. They were cleared from blood by use of a syringe flushing the efferent blood vessel with saline containing heparin (5000 U/l). The preparation of isolated gill arches was conducted according to Kreiss et al. 2015. Gill arches were suspended by their perfusion tubing attached to the chamber lid, while a magnetic stir bar within the chamber ensured constant mixing of the respiratory medium. The perfusion saline was prepared according to Holmgren and Nilsson (1974) with the exception of HCO_3_, which was calculated after Heisler (1984, 1986) according to Kreiss et al. 2015 (10 °C, 550 µatm *P*CO_2_: 11.50 mM; 10 °C, 1200 µatm *P*CO_2_: 14.00 mM; 10 °C, 2200 µatm *P*CO_2_: 16.18 mM; 18 °C, 550 µatm *P*CO_2_: 7.11 mM; 18 °C, 1200 µatm *P*CO_2_: 8.63 mM; 18 °C 2200 µatm *P*CO_2_: 10.00 mM).

The pH of the Ringer solution was adjusted by a computerized feedback system using gaseous CO_2_ (Aqua medic GmbH Bissendorf, Germany) to 7.95 ± 0.05 for treatments at 10 °C and to 7.88 ± 0.07 for treatments at 18 °C. Seawater for respiration measurements was equilibrated with a membrane pump (Schemel & Goetz & Co KG, Offenbach, Germany) and reached pH = 8.09 ± 0.06 at 10 °C and low *P*CO_2_ and pH = 8.09 ± 0.06 at 18 °C and low *P*CO_2_. Equilibration with defined gas mixtures (AGA gas AB, Sweden) led to pH values matching the desired levels of *P*CO_2_ (10 °C, medium (1200 µatm): 7.74 ± 0.01; 10 °C, high (2200 µatm): 7.52 ± 0.02; 18 °C, medium: 7.75 ± 0.01; 18 °C, high: 7.55 ± 0.03).

### Energy budget of branchial ion transporters

Gill respiration was analysed according to Kreiss et al. (2015). In brief, the oxygen uptake of isolated perfused gill arches from the respiratory medium (seawater with incubation equivalent CO_2_ levels) was measured in thermostated (10 or 18 °C) chambers, by use of oxygen micro-optodes (needle type, 140 µm, PreSens, Regensburg, Germany). Fractional respiratory costs of branchial ion transporters were investigated via inhibitors for Na^+^/K^+^-ATPase (ouabain), H^+^-ATPase (bafilomycin A1), Na^+^/H^+^-exchanger {[5-(*N*-ethyl-*N*-isopropyl) amiloride] (EIPA)} and HCO_3_^−^-transporter [4,4′-Diisothiocyano-2,2′stilbenedisulfonic acid (DIDS)] applied to the respiratory medium following earlier methodological improvements (Kreiss et al. 2015). Transport inhibitors (obtained from Applichem, Darmstadt, Germany or Sigma-Aldrich, Taufkirchen, Germany) were dissolved in DMSO at a final DMSO concentration of less than 1 %, which had no effect on respiration in control experiments (data not shown). Final concentrations of inhibitors were in the range of literature values: ouabain 5 mM (Krumschnabel and Wieser 1994; Mark et al. 2005) bafilomycin A1 0.1 µM (Morgan and Iwama 1999; Pörtner et al. 2000), EIPA 80 µM (Wu et al. 2010 (100 µM)) and DIDS 1 mM (Duraton et al. 1997; Parks et al. 2007). Each gill arch was used as its own control prior to application of one inhibitor. Control and inhibitor experiments were conducted for a period of 45 min each while recording oxygen consumption. After experimentation, the gill arches were separated from tubing and blotted dry for determining their total weight. Soft tissue was isolated and weighed after cutting as close as possible to the arch. For each of the six treatments and for each inhibitor, a set of ten gill arches from ten individual fish (five per replicate tank) were measured. Oxygen consumption rates were normalised to soft tissue weight (without the cartilaginous arch) as M(O_2_) = [µmol (O_2_) × gFW^−1^ × h^−1^].

### Osmolality and ion composition

Blood was centrifuged for 10 min at 1000*g* and 4 °C. Osmolality was measured in the resultant plasma with a Vapour Pressure Osmometer (Wescor Inc., Utah, USA). Ion composition was determined chromatographically. Na^+^ concentration was analysed amongst other cations by use of an Ion Chromatography System (DIONEX-ICS 2000, CA, USA) at 40 °C including an IonPac CS 16 column, with methane sulfonic acid (30 mM) as eluent at 0.36 ml min^−1^ flow rate. Cation concentrations were calculated according to a cation standard (Dionex, Six Cation Standard, CA, USA). Anions were separated on an Ion Pac AS11HC column with potassium hydroxide (30 mM, flow rate 0.30 ml min^−1^) as an eluent. Dionex Five Anion Standard was used as a reference to calculate anion concentrations. All ion concentrations are presented as mM. Plasma total CO_2_ (CCO_2_) of two treatment groups [10 °C, medium *P*CO_2_ (*n* = 4); 18 °C, high *P*CO_2_, (*n* = 46)] was determined with a carbon dioxide analyser (Corning 965, CIBA, Corning diagnostics, UK). Plasma bicarbonate levels were calculated from total CO_2_ by subtracting physically dissolved CO_2_ adopting ambient *P*CO_2_ levels.

### Data analysis and statistics

Statistical analysis was performed using Sigma plot 12.0 and Graphpad Prism 4. Seawater chemistry was tested for differences between *P*CO_2_, temperature and replicate tanks performing repeated measures two-way ANOVAs with subsequent Sidak’s multiple comparison tests (Table 2). All data are depicted as mean ± standard error. Outliers at a 99 % confidence level were identified using Nalimov’s test and removed from the plasma data set. Means of plasma ion concentrations, SMR, net O_2_ demands of isolated perfused gills and of HCO_3_^−^-transporter were compared using two-way ANOVAs with subsequent Tukey multiple comparison tests (Table 3). Osmolality, net O_2_ demand determined in the remaining isolated gill inhibitor experiments (of Na^+^/K^+^-ATPase, Na^+^/H^+^-exchanger, H^+^-ATPase) as well as the fractional weight of gill soft tissue were not normally distributed and therefore compared via one-way ANOVAs or Kruskal–Wallis non-parametric ANOVAs per temperature (Table 4). To test for differences between CO_2_ concentrations within these datasets, subsequent unpaired *t* tests with Holm-Bonferroni correction was used. Gill total respiration as the fraction of standard metabolic rate of whole fish (%) was compared within treatments at 10 °C performing a one-way ANOVA, while Holm-Bonferroni corrected unpaired *t* tests were conducted to detect differences between all treatments. Differences between remaining treatments were also tested via unpaired *t* tests except for the two low *P*CO_2_ groups where only means of gill and whole animal respiration could be compared for low *P*CO_2_ fish at 18 °C, due to low *n* numbers at 18 °C. A significance level of *p* < 0.05 was adopted for all data. *Q*_10_ values were calculated for the mean net oxygen demand of the four transporters investigated in the groups exposed to 10 and 18 °C and the respective *P*CO_2_ levels. Unfortunately, fish from the two tanks at 18 °C and low *P*CO_2_ could not be clearly assigned to their original tank as these were accidently mixed after respiration experiments. For the remaining groups, we performed *t* tests to detect potential differences between replicates. Except for the standard metabolic rates of the two groups at low *P*CO_2_ and 18 °C (see above) no differences in any of the tested parameters were detected between fish groups from replicate tanks.

**Table 2:** Results of repeated measures two-way ANOVAs performed to investigate potential differences between acclimation conditions (*P*CO_2_; temperature levels) and replicate tanks within and between these treatments (low *P*CO_2_ = 550 µatm; medium *P*CO_2_ = 1200 µatm and high *P*CO_2_ = 2200 µatm at 10 °C; 18 °C)

Variable	*P*CO_2_ effect			Temperature effect			Replicate effect			Interaction		
	*F*	*dF*	*P*	*F*	*dF*	*P*	*F*	*dF*	*P*	*F*	*dF*	*P*
*P*CO_2_	1446	2	**<0.0001**	–	–	–	6.208	3	0.007	2.241	6	0.045
Temperature	–	–	**–**	39.060	1	**<0.0001**	0.4647	1	0.4970	0.0064	1	0.6951
Low *P*CO_2_	–	–	–	34.61	1	**<0.0001**	0.0864	1	0.7709	1.251	1	0.2729
Medium *P*CO_2_	–	–	**–**	26.29	1	**<0.0001**	0.0008	1	0.9765	0.7693	1	0.3879
High *P*CO_2_	–	–	–	1.593	1	0.5697	1.067	1	0.3105	0.5697	1	0.3309
10 °C	307.8	2	**<0.0001**	–	–	–	0.2700	1	0.6115	0.0385	2	0.9623
18 °C	352.9	2	**<0.0001**	–	–	–	0.462	1	0.5078	1.838	2	0.1778

Significant differences are presented in bold

**Table 3:** Results of two-way ANOVAs performed to investigate potential differences of SMR, plasma ion concentrations and gill oxygen consumption between differently exposed fish groups (low *P*CO_2_ = 550 µatm; medium *P*CO_2_ = 1200 µatm and high *P*CO_2_ = 2200 µatm at 10 °C; 18 °C)

Variable	*P*CO_2_ effect			Temperature effect			Interaction		
	*F*	*dF*	*P*	*F*	*dF*	*P*	*F*	*dF*	*P*
SMR	0.0931	2	0.911	16.36	1	**0.0002**	2.383	2	0.1025
Plasma Na^+^	0.318	2	0.729	19.298	1	**<0.001**	6.535	2	**0.003**
Plasma Cl^−^	3.803	2	**0.027**	3.642	1	0.060	9.024	2	**<0.001**
Gill MO_2_	0.9367	2	0.393	89.33	1	**<0.0001**	0.1381	2	0.8711
HCO_3_^−^ -transporter	7.871	2	**0.001**	4.297	1	**0.043**	2.287	2	0.112

Significant differences are presented in bold

**Table 4:** Results of one-way ANOVAs, respectively, Kruskal–Wallis one-way ANOVAs performed to investigate potential differences of osmolality and ion transporter net O_2_ demand from isolated gill experiments between differently exposed fish groups (low *P*CO_2_ = 550 µatm; medium *P*CO_2_ = 1200 µatm and high *P*CO_2_ = 2200 µatm) at 10 °C, respectively, 18 °C

Variable	10 °C			18 °C		
	*F*	*dF*	*P*	*F*	*dF*	*P*
Osmolality	0.02401	2	0.9763	6.775	2	**0.0032**
Na^+^/K^+^-ATPase	0.878	2	0.428	H = 1.355	2	0.508
H^+^-ATPase	0.347	2	0.710	0.592	2	0.560
Na^+^/H^+^-exchanger	4.025	2	**0.030**	6.064	2	**0.007**
% Gill filament weight	H = 14.96	2	**<0.001**	H = 1.561	2	0.458

*H* value instead of *F* value is given when non-parametric Kruskal–Wallis test was performed

Significant differences are presented in bold

## Results

### Seawater chemistry and mortality

Seawater chemistry and temperatures of the 12 tanks are summarised in Table 1. *P*CO_2_ and temperature differed significantly (*p* < 0.0001) between tanks according to experimental conditions (Table 2). Statistical interactions found for *P*CO_2_ replicates are explained by differences between data measured at 10 and 18 °C. In general, *P*CO_2_ values calculated for 18 °C were above those found at 10 °C (Table 1). This was especially pronounced for the low and medium *P*CO_2_ groups (Table 2), however, the difference of approximately 100 µatm was considered negligible from a physiological view. Tank replicates did not differ between any treatment neither for temperature nor for *P*CO_2_ levels (Table 2). For *P*CO_2_ treatments at 10 and 18 °C, replicates were maximally ±0.5° different (*p* < 0.0001). This difference might be explained by a slight temperature gradient within the rooms and was considered minor.

Total mortality during incubation was 8.2 % (nine fish); however, almost half of the casualties (four fish) were found at 18 °C and low *P*CO_2_, while other losses were evenly distributed between exposure groups. Furthermore, two fish died of asphyxiation (see above) and one animal died from the consequences of jumping out of the tank, leaving us with a total of 99 fish.

### Oxygen consumption of whole animals

**Fig. 1:**
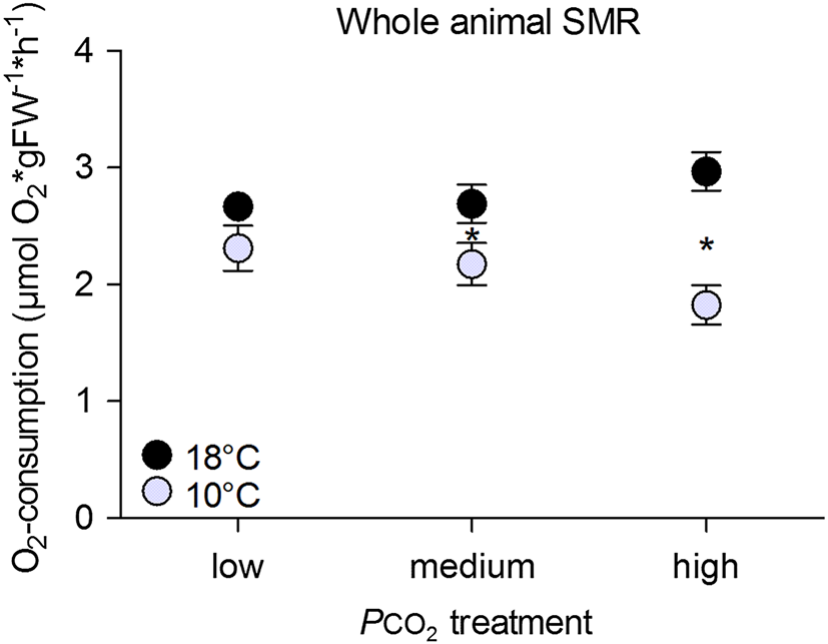
Standard metabolic rate (µmol O_2_ × gFW^−1^ × h^−1^) of cod 4 weeks exposed to low *P*CO_2_ = 550 µatm (L), medium *P*CO_2_ = 1200 µatm (M) and high *P*CO_2_ = 2200 µatm (H) at 10 °C (*grey circles*) and at 18 °C (*black circles*). *n* = 4–12 per treatment. Symbols indicate significant differences between temperature groups (*p* < 0.05)

Mean standard metabolic rates related to body mass were in the same range for fish reared at low *P*CO_2_ and both incubation temperatures (10 °C: 2.31 ± 0.64 µmol O_2_ × gFW^−1^ × h^−1^; 18 °C: 2.67 ± 0.04 µmol O_2_ × h^−1^ × gFW^−1^) (Fig. 1). Increasing CO_2_ levels influenced metabolic rate differently depending on incubation temperature (Fig. 1). Significant differences between groups at the two temperatures were observed at either medium or high *P*CO_2_ when the fish reared at 18 °C displayed a higher SMR than those acclimated to 10 °C (*p* = 0.043 at medium *P*CO_2_; *p* < 0.0001 at high *P*CO_2_).

### Plasma osmolality and ion concentrations

**Fig. 2:**
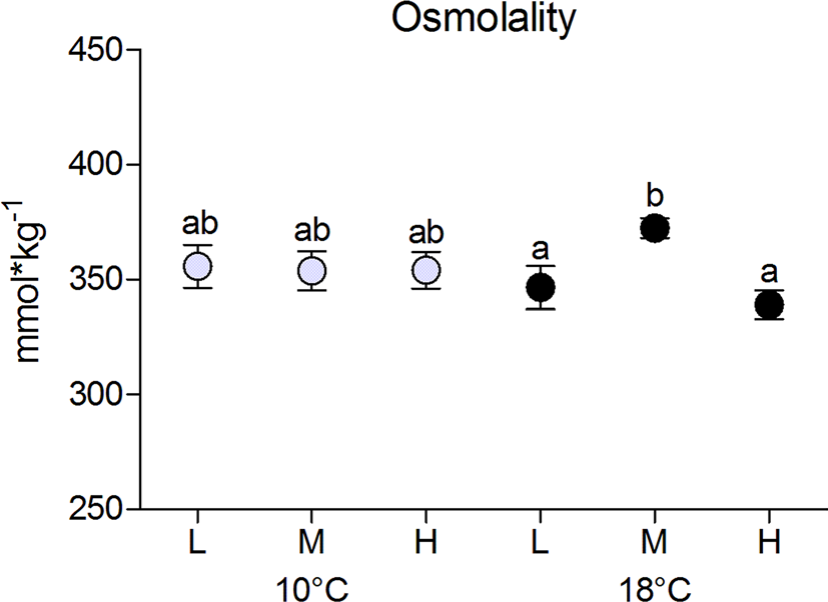
Plasma osmolality (mmol kg^−1^) of cod 4 weeks exposed to low *P*CO_2_ = 550 µatm (L), medium *P*CO_2_ = 1200 µatm (M) and high *P*CO_2_ = 2200 µatm (H) at 10 °C (*grey circles*) and at 18 °C (*black circles*). *n* = 18–20 per treatment. *Different letters* indicate significant differences between treatment groups (*p* < 0.05)

**Fig. 3:**
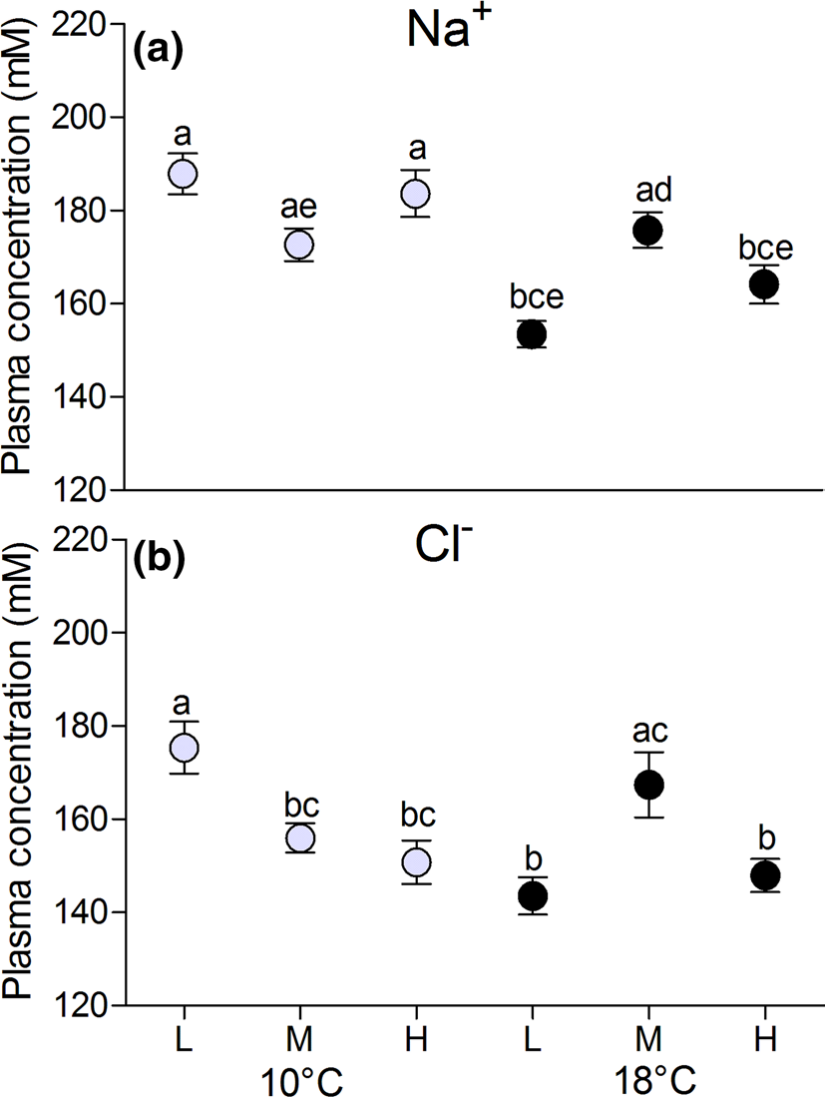
**a**, **b** Blood plasma Na^+^ and Cl^−^ concentrations (mM) in plasma of cod 4 weeks exposed to low *P*CO_2_ = 550 µatm (L), medium *P*CO_2_ = 1200 µatm (M) and high *P*CO_2_ = 2200 µatm (H) at 10 °C (*grey circles*) and at 18 °C (*black circles*). *n* = 18–20 per treatment. *Different letters* indicate significant differences between treatment groups (*p* < 0.05)

Plasma osmolality (Fig. 2) was independent of CO_2_ in cod reared long term at 10 °C (optimum temperature, total mean 354.53 ± 31.52 mmol kg^−1^). It was significantly lower in cod at 18 °C under low and high CO_2_ compared to fish reared at 18 °C and medium *P*CO_2_ (*p* < 0.05). No statistical temperature effect was detected between fish reared at 10 °C and 18 °C. Plasma ion concentrations (Fig. 3a, b) at low *P*CO_2_ and 10 °C were in the range of 188 ± 16 mM for Na^+^, and 175 ± 21 mM for Cl^−^ (Fig. 3a, b). For fish at 10 °C, both ions were found at ~10 % lower concentrations at medium *P*CO_2_ than under low *P*CO_2_ (*p* = 0.057 Na^+^, *p* = 0.025 Cl^−^). At 10 °C and high *P*CO_2_, Cl^−^ concentrations fell below values of animals at low *P*CO_2_ (15 %; *p* = 0.004); whereas Na^+^ plasma levels were only marginally reduced (3 %). Plasma bicarbonate for the medium *P*CO_2_ group at 10 °C was in the range of 10.99 ± 0.69 mM.

Warming to 18 °C under low *P*CO_2_ led to a ~20 % reduction in the concentrations of both ions (*p* = 0.012, Fig. 3a, b). At 18 °C and medium *P*CO_2_, Na^+^ and Cl^−^ concentrations were less distinct from the group at 10 °C and low *P*CO_2_. This pattern was not observed at high *P*CO_2_ and 18 °C such that plasma ion levels at 18 °C were reduced in fish under low and high CO_2_ levels compared to medium *P*CO_2_ (Na^+^: low *P*CO_2_*p* = 0.002, high *P*CO_2_*p* = 0.004; Cl^−^: low *P*CO_2_*p* = 0.002, high *P*CO_2_*p* = 0.006) (Fig. 3a, b). For animals from all treatments, mean plasma Na^+^ concentration exceeded that of Cl^−^ by 14–31 mM positive charges. Plasma bicarbonate for the high *P*CO_2_ group at 18 °C was in the range of 9.81 ± 1.68 mM.

### Gill fresh weight

Mean gill fresh weight determined per single arch after gill respiration experiments was CO_2_ dependent at 10 °C. Soft gill tissue and thereby also total gill weight increased in fish at 10 °C and medium or high *P*CO_2_ (*p* < 0.05) compared to the group at 10 °C and low *P*CO_2_ (Table 5). This pattern was also mirrored in the fraction of gill soft tissue weight (related to whole animal weight) and thereby independent of an allometric effect (Table 5). At high temperature, no CO_2_ effect was observed. Weights of total gill and gill soft tissue for the three groups at 18 °C were in the range of those from low *P*CO_2_ fish at 10 °C, being significantly lower than the size-increased gills in fish at 10 °C and medium or high *P*CO_2_ treatments.

**Table 5:** Weight of total gill, respectively, gill soft tissue without cartilaginous arch, as well as fractional soft tissue weight related to whole animal weight of the differently exposed groups (low *P*CO_2_ = 550 µatm; medium *P*CO_2_ = 1200 µatm and high *P*CO_2_ = 2200 µatm at 10 °C; 18 °C) given as mean ± standard deviation, *n* = 40 per treatment for total gill and soft tissue weight, *n* = 3–8 for fractional (%) gill weight

weight (g)	10 °C low	10 °C medium	10 °C high	18 °C low	18 °C medium	18 °C high
Gill arch	0.52 ± 0.15a	0.65 ± 0.25bc	0.69 ± 0.25b	0.56 ± 0.25ac	0.57 ± 0.21ac	0.52 ± 0.20a
Gill soft tissue	0.28 ± 0.08a	0.36 ± 0.13bc	0.38 ± 0.15b	0.26 ± 0.13a	0.29 ± 0.11ac	0.26 ± 0.12a
% Weight	0.13 ± 0.07a	0.27 ± 0.22b	0.18 ± 0.11bc	0.17 ± 0.14ab	0.12 ± 0.04a	0.16 ± 0.10ab

### Gill respiration and energy budget of ion regulation transporters

**Fig. 4:**
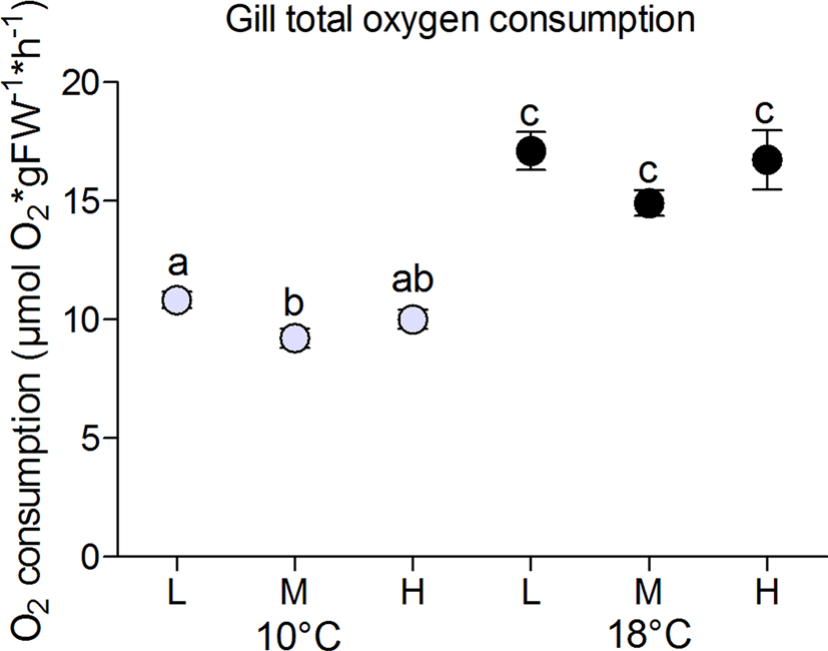
Gill total oxygen consumption (µmol O_2_ × gFW^−1^ × h^−1^) of cod 4 weeks exposed to low *P*CO_2_ = 550 µatm (L), medium *P*CO_2_ = 1200 µatm (M) and high *P*CO_2_ = 2200 µatm (H) at 10 °C (*grey circles*) and at 18 °C (*black circles*). *n* = 40 per treatment. *Different letters* indicate significant differences between treatment groups (*p* < 0.05)

Mean oxygen consumption related to gill soft tissue was 10.8 µmol O_2_ × gFW^−1^ × h^−1^ at 10 °C and low *P*CO_2_, whereas gills from fish reared at 10 °C and medium *P*CO_2_ displayed respiration rates about 15 % lower than those at low *P*CO_2_ (*p* = 0.013) (Fig. 4). Gill oxygen consumption rates at high *P*CO_2_ and 10 °C were found between those at the other *P*CO_2_ levels, but did not differ significantly from those. Fish acclimated to 18 °C had higher branchial respiration rates than those reared at 10 °C (*p* < 0.001) (Fig. 4), following a *Q*_10_ of 1.8 ± 0.07. Gill oxygen consumption rates at the three *P*CO_2_ levels and 18 °C were not statistically different. Again, respiration rates were lowest at medium *P*CO_2_ as observed before at 10 °C (*p* = 0.057, Fig. 4).

**Fig. 5:**
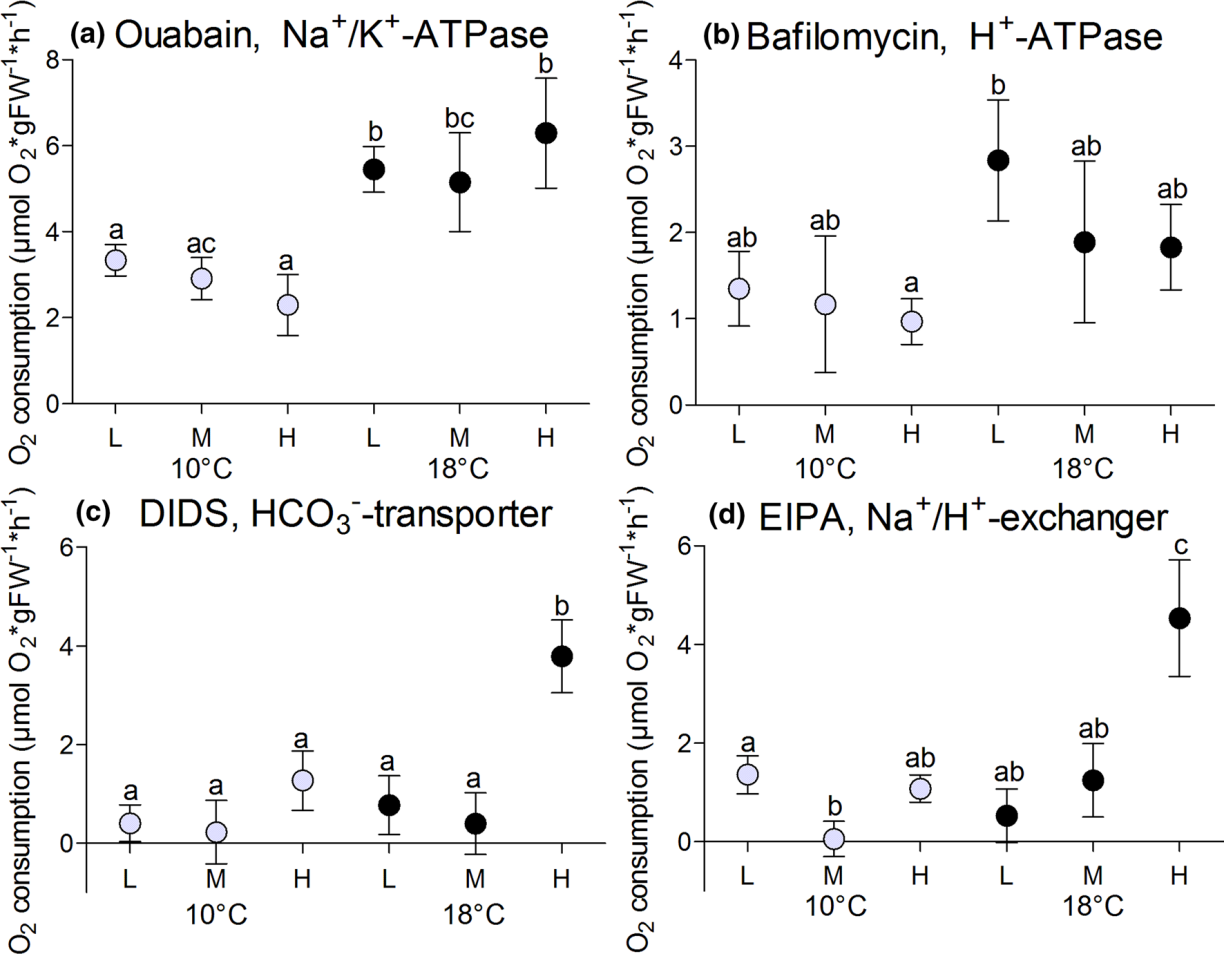
**a**–**d** Net O_2_ demand of cod gill Na^+^/K^+^-ATPase (**a**), H^+^-ATPase (**b**), HCO_3_^−^-transporter (**c**) and Na^+^/H^+^-exchanger (**d**) (µmol O_2_ × gFW^−1^ × h^−1^) of cod 4 weeks exposed to low *P*CO_2_ = 550 µatm (L), medium *P*CO_2_ = 1200 µatm (M) and high *P*CO_2_ = 2200 µatm (H) at 10 °C (*grey circles*) and at 18 °C (*black circles*), given as means with standard error of the mean. *Letters* indicate significant differences in net O_2_ demand of processes between treatment groups (*p* < 0.05), *n* = 10 per treatment

**Fig. 6:**
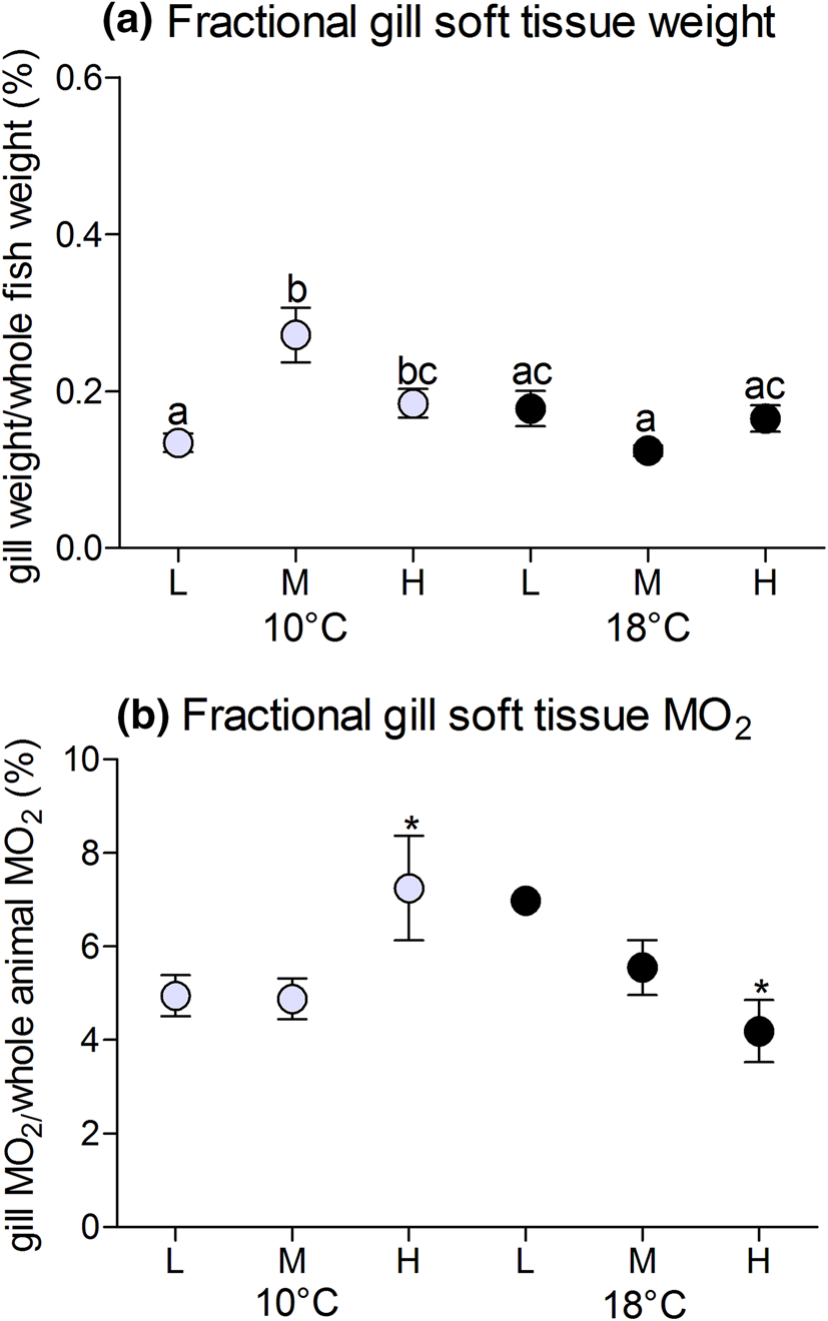
**a**, **b** Gill total respiration (**a**) and weight (**b**) as the fraction of standard metabolic rate, respectively, weight of whole animal 4 weeks exposed to low *P*CO_2_ = 550 µatm (L), medium *P*CO_2_ = 1200 µatm (M) and high *P*CO_2_ = 2200 µatm (H) at 10 °C (*grey circles*) and at 18 °C (*black circles*). *n* = 4–8 per treatment, for 18 °C low *P*CO_2_ a mean relation was calculated. *Letters* indicate significant differences in net O_2_ demand of processes between treatment groups (*p* < 0.05) (**a**); *symbols* indicate significant differences between treatments (**b**) as not all groups could be tested against each other (*p* < 0.05)

Net O_2_ demand of ion transporters, calculated as the difference between respiration rates of untreated and inhibitor-exposed gills, is presented in Fig. 6a–d; fractional costs in percent of total gill respiration are listed in Table 6. The inhibition of Na^+^/H^+^-exchanger (EIPA) and HCO_3_^−^-transporter (DIDS) occasionally led to respiration rates above controls, especially at medium *P*CO_2_ and 10 °C, indicating a compensatory increase in the activity of other transporters (Fig. 5c, d). For the transporters investigated, the largest fraction of branchial energy was claimed by Na^+^/K^+^-ATPase, at 3.3 ± 1.1 and 5.4 ± 1.7 µmol O_2_ × gFW^−1^ × h^−1^ at 10 and 18 °C, respectively (Fig. 5a), equivalent to 30 % of total gill respiration at low *P*CO_2_ conditions. This fraction remained independent of temperature at medium CO_2_ concentrations, however, a significantly elevated Na^+^/K^+^-ATPase fraction (38.80 %) was observed at 18 °C and high *P*CO_2_, larger than at high *P*CO_2_ and 10 °C (21.5 %) (*p* = 0.022) (Table 6). Oxygen demand by H^+^-ATPase (1.35 ± 1.30 µmol O_2_ × gFW^−1^ × h^−1^) and Na^+^/H^+^-exchanger (1.36 ± 1.21 µmol O_2_ × gFW^−1^ × h^−1^) at low *P*CO_2_ and 10 °C both comprised about 11 % of total gill respiration. HCO_3_^−^-transporter consumed about 3.7 ± 12.8 % (0.4 ± 1.2 µmol O_2_ × gFW^−1^ × h^−1^) (Fig. 5b–d; Table 6). At 18 °C, net O_2_ demand of Na^+^/K^+^-ATPase was elevated above rates seen in gills at 10 °C (*p* < 0.05), except when comparing the two medium *P*CO_2_ treatments which differed less (*p* = 0.098). O_2_-demand of H^+^-ATPase was also elevated at high temperature, but this effect became statistically significant only between gills at high *P*CO_2_ and 10 °C and those at low *P*CO_2_ and 18 °C (*p* = 0.023). CO_2_ dependent shifts in the usage of ion transporters were observed for Na^+^/H^+^-exchanger at both temperatures and for HCO_3_^−^ transport at 18 °C. Na^+^/H^+^-exchanger was reduced in terms of oxygen demand at medium *P*CO_2_ and 10 °C below rates at low *P*CO_2_ (*p* = 0.017) and rose at high *P*CO_2_ and 18 °C compared to gills of all other treatments (18 °C low *P*CO_2_ 7.6-fold, *p* = 0.017; 18 °C medium *P*CO_2_ 2.6-fold, *p* = 0.025; 10 °C low *P*CO_2_ 2.3-fold, *p* = 0.016; 10 °C medium *P*CO_2_ 82.8-fold *p* = 0.001, 10 °C high *P*CO_2_ 4.2-fold, *p* = 0.008). Mean absolute oxygen demand of HCO_3_^−^ transport was eightfold higher at high *P*CO_2_ and 18 °C than at medium *P*CO_2_ and 18 °C (*p* = 0.002) and at low *P*CO_2_ conditions and 10 °C (*p* = 0.0006), whereas oxygen demand of HCO_3_^−^ transport was increased fourfold at high *P*CO_2_ and 18 °C compared to low *P*CO_2_ gills at 18 °C (*p* = 0.007) (Fig. 5c).

**Table 6:** Cod gill fractional costs (%) of Na^+^/K^+^-ATPase, H^+^-ATPase, HCO_3_^−^ -transporter and Na^+^/H^+^-exchanger in the different exposed groups (low *P*CO_2_ = 550 µatm; medium *P*CO_2_ = 1200 µatm and high *P*CO_2_ = 2200 µatm at 10 °C; 18 °C) given as mean ± standard deviation

%	10 °C low	10 °C medium	10 °C high	18 °C low	18 °C medium	18 °C high
Na^+^/K^+^-ATPase	29.95 ± 5.64 ab	28.60 ± 10.99ab	21.53 ± 18.77b	29.95 ± 8.53ab	30.65 ± 14.83ab	38.8 ± 11.04ac
H^+^-ATPase	10.37 ± 10.45	6.23 ± 21.89	8.70 ± 7.66	14.81 ± 8.84	11.25 ± 19.16	11.18 ± 9.21
HCO_3_^−^-transporter	3.68 ± 12.84ab	1.79 ± 20.81ab	11.33 ± 17.59ab	4.93 ± 10.47ac	3.59 ± 11.25ad	23.57 ± 12.21b
Na^+^/H^+^-exchanger	11.37 ± 9.59ab	-3.48 ± 17.06ab	10.64 ± 7.80 ab	3.67 ± 12.43ac	7.47 ± 14.19ad	19.28 ± 8.46b

Different letters indicate significant differences in fractional costs of processes between treatments (*p* < 0.05), *n* = 8 per treatment

In gills at 18 °C compared to those at 10 °C and the same level of *P*CO_2_ warming led to a strong *P*CO_2_ effect as indicated by elevated net O_2_ demand and an increase in the respective *Q*_10_ values (Fig. 5a–d; Table 7). Three of the four transporters investigated had a significantly higher net O_2_-demand at high *P*CO_2_: Na^+^/K^+^-ATPase, (*p* = 0.013), Na^+^/H^+^-exchanger, (*p* = 0.008), HCO_3_^−^-transporter, (*p* = 0.016). The related *Q*_10_ values for transporter activity between 10 and 18 °C under high *P*CO_2_ ranged between 2.6 and 5.8 (Table 7).

**Table 7:** *Q*_10_ values calculated for the mean net oxygen demand of the four transporters investigated between 10 and 18 °C groups at the respective *P*CO_2_ levels

*Q*_10_	Low *P*CO_2_	Medium *P*CO_2_	High *P*CO_2_
Na^+^/K^+^-ATPase	1.78	2.05	3.78
H^+^-ATPase	1.93	1.80	2.58
HCO_3_^−^-transporter	0.88	0.65	3.18
Na^+^/H^+^-exchanger	0.32	29.68	5.81

## Discussion

Atlantic cod reared under various levels of *P*CO_2_ and temperature responded by adjustments in branchial ion regulation and associated costs with implications for whole animal oxygen demand, osmolality, and Na^+^ and Cl^−^ concentrations in the plasma.

### Whole animal respiration

Standard metabolic rate (SMR) of cod (mean weight 193.34 ± 62.4 g) at 10 °C and low *P*CO_2_ (2.3 ± 0.6 µmol O_2_ × gFW^−1^ × h^−1^ or 74.0 ± 20.6 mg O_2_ × kg^−1^ × h^−1^) compare well with values published for 200 g Atlantic cod reared at 10 °C, ranging from 55–121 mg O_2_ × kg^−1^ × h^−1^ (Schurmann and Steffensen 1997). The potential impact of elevated *P*CO_2_ on aerobic scope and metabolic rates have been assessed in a number of fish species from the tropics to the Antarctic with the general hypothesis that the cost of coping with elevated *P*CO_2_ (acid–base and osmoregulation as well as cardiorespiratory adjustments) would increase SMR and/or cause a shift in energy budget and reduce aerobic scope and finally fitness (cf. Heuer and Grosell 2014). In Atlantic cod at 10 °C, SMR remained unaffected by elevated ambient *P*CO_2_. At low *P*CO_2_, SMR was compensated for during long-term warm exposure leading to similar rates in 10 and 18 °C animals (2.67 ± 0.04 µmol O_2_ × h^−1^ × gFW^−1^ or 85.38 ± 1.3 mg O_2_ × kg^−1^ × h^−1^), similar to those in Atlantic cod from Øresund, near to our study location, after thermal acclimation for several months to 10 and 15 °C (Schurmann and Steffensen 1997). Such compensation for changing temperatures is in line with earlier findings that cod can undergo thermal acclimation and thereby endure warm periods. Despite the down-regulation of SMR during warm acclimation, branchial respiration rates were enhanced at 18 °C (all treatments) indicating a shift in energy budget (see above) possibly constraining residual aerobic scope (cf. Pörtner et al. 2010). Further careful analysis of various components of aerobic energy budget and their trade-offs under elevated CO_2_ levels is thus warranted. In line with a high capacity of warm acclimatisation, wild southern North Sea cod remained for several weeks in their natural habitat at temperatures above 16 °C (Righton et al. 2010). In contrast, Atlantic cod from Scotlands’ west coast exposed to the same acclimation conditions as in the present study (10 and 18 °C for 4 weeks) displayed a 70 % increase in routine metabolic rates during long-term warm exposure (Soofiani and Hawkins 1982), indicating that thermal acclimatisation capacity differs between cod populations (cf. Pörtner et al. 2008).

The response of SMR to increasing *P*CO_2_ levels was different at the two acclimation temperatures. While SMR at low *P*CO_2_ was similar due to effective compensation, SMR at 18 °C under medium and high *P*CO_2_ was higher than in fish reared under the same *P*CO_2_ levels at 10 °C (Fig. 1). Elevated *P*CO_2_ may thus offset the compensation under warming and cause an increase in metabolic cost and/or capacity. Both warming and acidification occur regularly in summer in the cod’s natural habitat with as yet unclear functional consequences.

### Gill maintenance costs

Oxygen consumption rates of gills isolated from Kattegat/Skagerrak cod reared at low *P*CO_2_ and 10 °C (5.81 ± 1.19 µatm O_2_ × gFW^−1^ × h^−1^) (Fig. 1) were in the range found in previous studies (Lyndon 1994; Deigweiher et al. 2010), but 50 % lower than those reported for Southern North Sea cod (Kreiss et al. 2015). Lower total gill oxygen demand might be attributed to seasonal changes or population differences.

Relating the weight and metabolic costs of soft gill tissue to the weight of the whole fish (Fig. 6a, b) revealed an interesting pattern. The fraction of soft gill tissue was significantly elevated in medium and high *P*CO_2_ fish at 10 °C compared to fish reared at low *P*CO_2_ and 10 °C (twofold at medium *P*CO_2_, 1.4-fold at high *P*CO_2_; *p* < 0.001) (Fig. 6a). Such an increase in gill soft tissue mass in relation to whole animal weight may indicate branchial remodelling, a mechanism contributing to altered gill mass. Gill remodelling has also been reported in freshwater fish in response to various environmental factors (Goss et al. 1998; Nilsson 2007; Mitrovic and Perry 2009), and is supported by results from Atlantic halibut, where elevated *P*CO_2_ for 4 months caused an upregulation of proteins related to cellular turnover in the gill soft tissue (de Souza et al. 2014). A potentially higher total oxygen demand of larger gill arches will likely impact whole animal energy budget. Under low and medium *P*CO_2_ at 10 °C the fractional oxygen consumption of gills accounted for approx. 5 % of whole animal oxygen demand at rest, while this fraction increased with falling whole animal rates at high *P*CO_2_ reaching >7 % (*p* = 0.056) (Fig. 6b). For fish reared at 18 °C increasing *P*CO_2_ caused the fraction to decline with rising whole animal metabolic rates from about 7 % at low *P*CO_2_ to ~4 % at high *P*CO_2_. Taking into account that gill MO_2_ per gram soft tissue was maintained at high *P*CO_2_ and 10 °C (Fig. 4), we can postulate that branchial oxygen demand was increased due to the increase in tissue weight under this treatment (Fig. 6a) As gill oxygen consumption was stable in all *P*CO_2_ groups at 18 °C (Fig. 6a), higher demand for oxygen by other tissues would explain the reduced fractions of gill oxygen demand (Fig. 6b).

### Plasma osmolality and ion concentrations

At 10 °C, plasma osmolality was in the range of values reported earlier for cod and independent of ambient CO_2_ (Larsen et al. 1997; Herbert and Steffensen 2005). However, while osmolality remained constant at 10 °C, the concentration of Cl^−^ was reduced in fish under *P*CO_2_ ≥ 1200 µatm at 10 °C. At this temperature, plasma Na^+^ concentrations were only slightly reduced at medium *P*CO_2_ (*p* = 0.057) but not at high *P*CO_2_. This indicates that osmolality was balanced by other anions, and concomitantly raises the question which transport processes are responsible for the unequal reduction in Na^+^ and Cl^−^ plasma concentrations. Plasma bicarbonate levels, determined in fish at 10 °C and medium *P*CO_2_ compensated for the excess of positively charged Na^+^ over Cl^−^ up to a difference of ~6 mM, supporting earlier findings that HCO_3_^−^/Cl^−^ exchange takes place under hypercapnia (Larsen et al. 1997).

Warming close to maximum summer temperatures (18 °C) combined with hypercapnic conditions led to a different response in plasma osmolality and ion concentrations. All parameters showed non-linear “hump-backed-curves”, with reduced ion concentrations and slightly lowered osmolality in animals at 18 °C under low and high *P*CO_2_, while plasma parameters of animals at medium *P*CO_2_ and 18 °C were significantly higher (Figs. 2, 5a, b). The shape of these curves remains unexplained, but medium *P*CO_2_ levels and 18 °C are frequently experienced by cod in warm summers and preadaptation to this conditions may exist.

The reduction of ion concentrations and the slightly lowered osmolality of fish at 18 °C and low and high *P*CO_2_ reflects altered gill ion transporter activities indicated by enhanced fractions in oxygen demand. Net O_2_ demand of Na^+^/K^+^-ATPase and less so of H^+^-ATPase increased in the warmth (Fig. 5a, b). Thermally uncompensated (i.e. stimulated) in vivo activities of Na^+^/K^+^-ATPase and increased maximum transporter capacities (Na^+^/K^+^-ATPase and H^+^-ATPase) were also observed in the former study (Kreiss et al. 2015) implying that Na^+^/K^+^-ATPase might overcompensate for enhanced branchial permeability in the warmth. Together with the slightly reduced osmolality caused by the loss in ion concentration (Na^+^ and Cl^−^) in warm acclimated fish at low and at high *P*CO_2_ this indicates a shift in the electrochemical gradient with enhanced NaCl excretion against the inward ion gradient. Enhanced hypo-osmoregulation was reported before for Antarctic fish during warm acclimation (Gonzalez-Cabrera et al. 1995; Brauer et al. 2005). We assume that elevated transport capacities (*Q*_10_ effect) cause decreased osmolality despite higher leakage rates according to higher membrane fluidity. Thereby organic osmolytes may play a role in replacing inorganic osmolytes such as Na^+^ and Cl^−^ for the reasons mentioned above, but this compensation seems less effective for fish incubated at 18 °C under low and high *P*CO_2_ than observed in hypercapnic fish at 10 °C.

As the altered plasma ion concentration does not match the observed usage of branchial ion transport for all treatment groups (see below), further ion regulatory organs, such as kidney and the gastrointestinal tract may be involved in NaCl absorption using the same transporter types as found in branchial epithelia (Na^+^/K^+^-ATPase, Na^+^/K^+^/2Cl^−^ cotransporter). Anion including HCO_3_^−^ exchange (e.g. Cl^−^ and Na^+^ dependent HCO_3_^−^ exchanger) are also known to play an important role in the intestine and might cause altered plasma Na^+^ and Cl^−^ concentrations not explained by branchial processes (Marshall and Grosell 2005). For European flounder, reduced plasma osmolality was observed in response to experimentally enhanced intestinal bicarbonate excretion demonstrating that such alterations in the intestine can significantly impact whole animal osmolality status (Wilson et al. 2002).

### Gill ion regulation mechanisms in vivo

The fractions of Na^+^/K^+^-ATPase, H^+^-ATPase, Na^+^/H^+^-exchanger, and HCO_3_^−^-transporter in oxygen consumption comprise the activities of all isoforms of the respective transporter families. Na^+^/K^+^-ATPase (all isoforms) is located basolaterally (Evans et al. 2005). A basolateral location was also suggested for H^+^-ATPase in immunolocalization studies on dogfish (*Squalus acanthias*) and longhorn sculpin *Myoxocephalus octodecimspinosus*) (Tresguerres et al. 2005; Catches et al. 2006). Several isoforms were described for Na^+^/H^+^-exchange (e.g. Edwards et al. 2005; Deigweiher et al. 2008; Rimoldi et al. 2009) and HCO_3_^−^ transport (e.g. Piermarini et al. 2002; Esbaugh et al. 2012) located either apically or basolaterally.

The isolated tissue model applied here provides first insight into the affected processes, and can be better related to the whole animal level than isolated and reconstituted membrane models. When discussing the results, the apical or basolateral localisation of transporters from the same family should be kept in mind. While the inhibitors applied may have side-effects on other transporters, this did probably not have a large influence on our findings as the observed patterns of the applied inhibitors are in line with a specific mode of action on certain transporter (families). Under all conditions, O_2_ demand of Na^+^/K^+^-ATPase comprised about 30 % of total gill demand and thereby the largest fraction among the transporters investigated, followed by H^+^-ATPase and Na^+^/H^+^-exchanger (approx. 11 % each) and HCO_3_^−^-transport (approx. 3 %). This is in line with fractions of Na^+^/K^+^-ATPase and H^+^-ATPase in oxygen demand of cutthroat trout gill, which accounted for 37 % of total gill oxygen consumption (Morgan and Iwama 1999). Further studies examining fractions of oxygen allocation in isolated gills in vivo from marine fish found Na^+^/K^+^-ATPase to represent between 11.8 and 23.1 % of total gill respiration in two species of Notothenioidei (Deigweiher et al. 2010). The larger fractions of 43 % in cod (Kreiss et al. 2015) might again be explained by seasonal variation, as observed for other fish species (e.g. in yellow perch *Perca flavescens*, Packer and Garvin 1998). Differences between populations might involve various ion transporter activities and thereby altered total gill oxygen demand as found between the two cod studies.

Long-term exposure under high *P*CO_2_ is known to affect gill ion regulation capacity in teleosts involving increased mRNA levels and protein abundance of basolateral Na^+^/HCO_3_^−^ cotransporter and Na^+^/K^+^-ATPase, while those of anion exchanger (HCO_3_^−^/Cl^−^ transporter) and Na^+^/H^+^-exchanger remained at control levels (*Zoarces viviparus*, Deigweiher et al. 2008). In the present study, in vivo oxygen demand of these transporters at optimum temperature (10 °C) indicates a different pattern: The summed oxygen demand elicited by HCO_3_^−^-transporters as well as Na^+^/H^+^-exchangers indicates small responses to both hypercapnia levels applied. A slight enhancement of HCO_3_^−^-transporter activity occurred at high *P*CO_2_ level and the net O_2_ demand caused by Na^+^/H^+^-exchanger seen at the three *P*CO_2_ levels indicated minimum activity at medium but elevated activity under high *P*CO_2_ conditions. This indicates at least some involvement of both transporter types depending on the hypercapnia levels applied. Thereby, the interdependencies in the involvement of different transporters need to be considered, as in case of DIDS application to gills at 10 °C. DIDS caused a stimulation of oxygen consumption, possibly due to a shift to other, more costly transporters, which compensated for inhibited HCO_3_^−^-transport. Such a response has been observed before in isolated tissue of a marine worm (*Sipunculus nudus*) (Pörtner et al. 2000).

Slightly decreased in vivo Na^+^/K^+^-ATPase activities at high *P*CO_2_, are in line with earlier observations (Melzner et al. 2009; Esbaugh et al. 2012; Kreiss et al. 2015), where under *P*CO_2_ levels ≤3000 µatm, branchial Na^+^/K^+^-ATPase capacities remained unchanged or were reduced, contrasting the upregulation observed under high *P*CO_2_ ≥ 6000 µatm (Deigweiher et al. 2008, 2010; Melzner et al. 2009). The pathways eliciting these differences remain to be explored. H^+^-ATPase activity and its net O_2_ demand remained rather stable across CO_2_ treatments, indicating that the in vivo costs of proton excretion are not altered in the cod gill at optimum temperature after 4 weeks of exposure. In salmon, reduced levels of V-type H^+^-ATPase B-subunit mRNA were found under severe hypercapnia (20,000 µatm, Seidelin et al. 2001), again a certain *P*CO_2_ threshold for the response may be postulated for the H^+^-ATPase. Downregulation of this basolateral pump may cause an increase in branchial H^+^-excretion. Taken together, no clear molecular restructuring in ion transport was observed after 4 weeks of acclimation to prospective *P*CO_2_ at optimum temperature.

However, gill remodelling and the increase in gill soft tissue under medium and high *P*CO_2_ at 10 °C (Fig. 6a; Table 5; also found in Kreiss et al. 2015) may affect ion transporter usage. Freshwater fish, exposed to acute severe hypercapnia (~2 % CO_2_), displayed an increased branchial apical surface area of pavement cells, which reduced the surface area of exposed chloride cells and thereby modified the exchange rate of the apical HCO_3_^−^/Cl^−^ exchanger (Goss et al. 1998). The authors concluded that this mechanism reduces base-excretion and thus compensated for acidosis. As pavement cells have a lower oxygen demand than chloride cells the relative increment of pavement cells may explain the increase in soft tissue at constant oxygen consumption rate per g gill arch under hypercapnia. Whether these changes occurred and affected the physical exposure of chloride cells and transporters involved, needs to be confirmed by histological and mechanistic analyses. The absence of increased branchial soft tissue mass in the warmth could be prevented by the otherwise counterproductive reduction of the respiratory surface area.

At 18 °C and elevated *P*CO_2_, Na^+^/H^+^-exchanger and HCO_3_^−^ transporter both caused elevated O_2_ and energy demand compared to the respective rates at 10 °C. Na^+^/H^+^-exchanger elicited increased net O_2_ demand at both elevated *P*CO_2_ levels, whereas HCO_3_^−^-transport only did at high *P*CO_2_. Isoforms of Na^+^/H^+^-exchanger and HCO_3_^−^-transporter are localised either apically or basolaterally (cf. Heuer and Grosell 2014). Three isoforms of Na^+^/H^+^-exchangers (NHEs) are involved in fish acid–base regulation, NHE1 on the basolateral and NHE2 and NHE3 on the apical side (Evans et al. 2005; Deigweiher et al. 2008). Various transporters are involved in HCO_3_^−^-transport and are blocked by DIDS; the most important in terms of acid–base regulation might be the apical and basolateral HCO_3_^−^/Cl^−^ exchangers, called anion exchanger (AEs) and a Na^+^/HCO_3_^−^ co-transporter on the basolateral side (NBC1). At 18 °C, NHE costs were elevated in response to both hypercapnia treatments above those at low *P*CO_2_, indicating an upregulation of apical NHEs and enhanced acid excretion. In general, acid excretion in marine fish gills is thought to involve the thermodynamically favourable apical Na^+^/H^+^-exchanger more than the costly H^+^-ATPase (Claiborne et al. 1999; Tresguerres et al. 2005). A predominant role for apical Na^+^/H^+^-exchange in acid–base regulation was also found in gills of fresh and seawater adapted mummichogs (*Fundulus heteroclitus*) under acute, more severe hypercapnia (10,000 µatm) (Edwards et al. 2005), as well as during acid infusion (Claiborne et al. 1999). In this line, mRNA of the basolateral isoform of Na^+^/H^+^-exchanger was downregulated at least under short-term hypercapnia (1 h–4 days; Deigweiher et al. 2008; Rimoldi et al. 2009). In the present study, in vivo costs of both Na^+^/H^+^-exchanger and HCO_3_^−^-transport were enhanced under high CO_2_ levels. This pattern might involve a higher usage and expression of Na^+^/HCO_3_^−^-cotransporter NBC1 as observed before in gill tissues of eelpout (*Zoarces viviparus*) acclimated for 6 weeks to 10,000 µatm CO_2_ (Deigweiher et al. 2008).

Independent of the particular mechanism, our data support that either Na^+^/HCO_3_^−^-cotransporter or HCO_3_^−^/Cl^−^-exchanger, in parallel to Na^+^/H^+^-exchanger play a dominant role in cod acid–base regulation under moderate hypercapnia in the warmth. The observed stimulation of ion transporters at elevated *P*CO_2_ and 18 °C did not result in alterations of total gill oxygen consumption rates compared to low *P*CO_2_ at 18 °C. Stable total gill oxygen consumption despite shifted usage of ion transporter was observed before in Antarctic fish at 10,000 µatm CO_2_ (Deigweiher et al. 2010). The mechanistic background of this stability remains to be explored.

## Conclusions and perspectives

Whole animal standard metabolic rates of Atlantic cod incubated for 3 weeks were independent of temperature (10, 18 °C). These results confirm previous findings that Southern cod populations can acclimate and compensate for temperature, within limits marginally reached at 18 °C. With respect to the different CO_2_ concentrations applied (550, 1200 and 2200 µatm), these data also provide an evidence that near-term projected future *P*CO_2_ levels per se will have a small effect on SMR. Due to the changes in whole animal SMR between medium and high *P*CO_2_ groups of the two temperatures studied, we observed increasing fractions of gill in whole organism oxygen demand towards high *P*CO_2_ at 10 °C but decreasing fractions under rising *P*CO_2_ at 18 °C. This reflects increased overall gill oxygen consumption at 10 °C and high *P*CO_2_, whereas gills in fish at 18 °C displayed rather stable branchial oxygen demand that was not involved in fluctuations of cod energy turnover under CO_2_.

In parallel, our data provide evidence of increased demand for branchial ion regulation through Na^+^/H^+^-exchange and HCO_3_^−^-transport at 18 °C and elevated *P*CO_2_ leading to an associated reduction in inorganic plasma osmolytes via stimulated Na^+^/K^+^-ATPase and a shift in plasma ionic balance; the latter effect was also observed at low *P*CO_2_ and 18 °C. Reduced net O_2_ demand of Na^+^/H^+^-exchanger under medium *P*CO_2_ as well as the slight decrease of net Na^+^/K^+^-ATPase O_2_ demand under high *P*CO_2_ at 10 °C is an evidence for a different mechanism at optimum temperature than at 18 °C. To what extent these changes contribute to shifts in somatic energy turnover and budget, remains to be explored. Overall, Atlantic cod appear quite tolerant to elevated CO_2_ levels and variable temperatures, which may come with its demersal lifestyle and preadaptations to fluctuating CO_2_ levels and fluctuating temperatures in shallow water habitats.
